# Supplementary material for: Pedestrians' safety using projected time-to-collision to electric scooters
Source: Nat Commun. 2024 Jul 7;15:5701. doi: 10.1038/s41467-024-50049-x (PMC11228023; doi:10.1038/s41467-024-50049-x)
Supplement: Supplementary file 1 — Supplementary Information [file 41467_2024_50049_MOESM1_ESM.pdf]

Supplementary Tables

Pedestrians' safety using projected time-to-collision to electric scooters

Alireza Jafari and Yen-Chen Liu

Department of Mechanical Engineering, National Cheng Kung University

This appendix contains the statistical analysis results presented by box plots in Fig. 2. All the tests are done using Matlab 2023b. The following abbreviations are used in this appendix.
C13: Interaction setting C and width 1.3 m;
C16: Interaction setting C and width 1.6 m;
C20: Interaction setting C and width 2.0 m;
C24: Interaction setting C and width 2.4 m;
A30: Interaction setting A and width 3.0 m;
B30: Interaction setting B and width 3.0 m;
D13: Interaction setting D and width 1.3 m;
D20: Interaction setting D and width 2.0 m;
D30: Interaction setting D and width 3.0 m;
E20F: Interaction setting E and width 2.0 m for facing pedestrians;
E30F: Interaction setting E and width 3.0 m for facing pedestrians;
E20O: Interaction setting E and width 2.0 m for overtaking pedestrians.
E30O: Interaction setting E and width 3.0 m for overtaking pedestrians.

In Tables 2 to 7, power analysis is used to determine the sample size needed for a study to achieve a desired level of statistical power. In hypothesis testing, the statistical power, denoted by 1-β, represents the probability of correctly rejecting a null hypothesis when the alternative hypothesis is true considering the sample sizes.

Table 1 presents the details of each box plot including the whiskers, the quartiles, the notches, and the medians. In addition, four common normality tests examine the samples' normality. Furthermore, Tables 2–7 use Welch's test (two-way t-test for unequaled variances and sample sizes) to quantitatively demonstrate the significance of the difference in each subplot in Fig. 2, visually shown in the paper.

Table 2 contains Welch's test results for each pair of samples in Fig. 2(a), i.e., p-value and the confidence interval. The first sample is selected from the rows and the second sample is selected from the columns; the order affects the confidence interval sign since it shows the confidence interval on the difference of the population means. In addition, the power analysis result shows the power of the test considering the groups' sample sizes; the first value assumes rows as the base group and the second value assumes columns as the base.

Table 3 contains Welch's test results for each pair of samples in Fig. 2(b), i.e., p-value and the confidence interval. For each width, the first sample is an e-scooter facing a pedestrian and the second sample is an e-scooter facing an e-scooter; the order affects the confidence interval sign since it shows the confidence interval on the difference of the population means. In addition, the power analysis result shows the power of the test considering the groups' sample sizes; for the first value, the base group is an e-scooter facing a pedestrian and for the second value, it is an e-scooter facing an e-scooter.

Table 4 contains Welch's test results for each pair of samples in Fig. 2(c), i.e., p-value and the confidence interval. For a width of 3.0 m, the first sample is an e-scooter facing a pedestrian and the second sample is an e-scooter overtaking a pedestrian; the order affects the confidence interval sign since it shows the confidence interval on the difference of the population means. In addition, the power analysis result shows the power of the test considering the groups' sample sizes; for the first value, the base group is an e-scooter facing a pedestrian and for the second value, it is an e-scooter overtaking a pedestrian.

Table 5 contains Welch's test results for each pair of samples in Fig. 2(d), i.e., p-value and the confidence interval. For each width, the first sample is an e-scooter facing a pedestrian and the second sample is an e-scooter moving through an arbitrary crowd when the facing pedestrians are picked; the order affects the confidence interval sign since it shows the confidence interval on the difference of the population means. In addition, the power analysis result shows the power of the test considering the groups' sample sizes; for the first value, the base group is an e-scooter facing an isolated pedestrian and for the second value, it is an e-scooter facing a pedestrian in a crowd.

Table 6 contains Welch's test results for each pair of samples in Fig. 2(d), i.e., p-value and the confidence interval. For isolated and crowd trials, the first sample is an e-scooter moving on a sidewalk with 2.0 m width and the second sample is with 3.0 m; the order affects the confidence interval sign since it shows the confidence interval on the difference of the population means. In addition, the power analysis result shows the power of the test considering the groups' sample sizes; for the first value, the base group is an e-scooter moving on a sidewalk with a 2.0 m width, and for the second value, it is an e-scooter moving on a sidewalk with a 3.0 m width.

Table 7 contains Welch's test results for each pair of samples in Fig. 2(e), i.e., p-value and the confidence interval. For a width of 3.0 m, the first sample is an e-scooter overtaking a pedestrian and the second sample is an e-scooter moving through an arbitrary crowd when the overtaken pedestrians are picked; the order affects the confidence interval sign since it shows the confidence interval on the difference of the population means. In addition, the power analysis result shows the power of the test considering the groups' sample sizes; for

the first value, the base group is an e-scooter overtaking an isolated pedestrian and for the second value, it is an e-scooter overtaking a pedestrian in a crowd.

Table 1: Box plots detail and normality tests for the datasets. The null hypothesis is that the set is normal with a 95% confidence level. The test result is 1 when it rejects the null hypothesis and 0 when it fails to reject. Rows are title, sample size, upper whisker, upper quartile, upper notch, median, lower notch, lower quartile, lower whisker, KS limiting form, KS Marsaglia, Shapiro-Wilk, and D'Agostino-Pearson, respectively.

Title	C13	C16	C20	C24	A30	B30	D13	D20	D30	E20F	E30F	E20O	E30O
Sample	30	30	30	30	100	100	30	30	30	28	27	22	23
Up. Wh.	0.46	0.57	0.52	0.79	0.82	2.16	0.3	0.3	0.37	0.87	0.83	1.42	1.65
Up. Q.	0.4	0.4	0.44	0.57	0.6	1.48	0.26	0.3	0.27	0.6	0.56	1.22	1.23
Up. Notch	0.38	0.35	0.41	0.53	0.54	1.32	0.26	0.29	0.25	0.5	0.48	1.14	1.11
Median	0.37	0.31	0.38	0.49	0.52	1.24	0.25	0.28	0.22	0.45	0.42	1.04	0.98
Low. Notch	0.35	0.26	0.36	0.44	0.49	1.16	0.23	0.27	0.2	0.39	0.35	0.93	0.84
Low. Q.	0.34	0.25	0.35	0.39	0.45	0.99	0.23	0.27	0.2	0.4	0.35	0.91	0.86
Low. Wh.	0.3	0.16	0.28	0.27	0.3	0.55	0.21	0.23	0.18	0.33	0.26	0.76	0.54
KS Lim.	1	1	1	1	1	1	1	1	1	1	1	1	1
KS Mars.	1	1	1	1	1	1	1	1	1	1	1	1	1
Shap.-W.	1	1	0	1	0	1	1	1	1	1	0	0	0
DAgos.	1	1	0	1	1	1	1	1	1	1	0	0	1

Table 2: Welch's test (two-way t-test for unequaled variances and sample sizes) results for each pair of boxplots in Fig. 2(a): p-value and Confidence Interval (C.I.). The test power, 1-β, for the given sample sizes of the two groups, is also presented. p-value smaller than 0.001 is shown by 0^+.

Sample	C13	C16	C20	C24
C16	p=0.013,<br>1-β= 0.50, 1.00,<br>C.I.=[-0.088 -0.011].			
C20	p=0.028,<br>1-β=0.4, 0.99,<br>C.I.=[0.005 0.081].	p=0^+,<br>1-β=0.96, 0.96,<br>C.I.=[0.043 0.142].		
C24	p=0^+,<br>1-β=0.92, 1.0,<br>C.I.=[0.067 0.169].	p=0^+,<br>1-β=1.0, 1.0,<br>C.I.=[0.107 0.227].	p=0.015,<br>1-β=0.58, 0.85,<br>C.I.=[0.015 0.135].	
A30	p=0^+,<br>1-β=1.0, 1.0,<br>C.I.=[0.131 0.189].	p=0^+,<br>1-β=1.0, 1.0,<br>C.I.=[0.166 0.253].	p=0^+,<br>1-β=0.99, 1.0,<br>C.I.=[0.074 0.160].	p=0.133,<br>1-β=0.35, 0.32,<br>C.I.=[-0.013 0.097].

Table 3: Welch's test (two-way t-test for unequaled variances and sample sizes) results when an e-scooter faces a pedestrian versus when it faces an e-scooter, boxplots in Fig. 2(b): p-value and Confidence Interval (C.I.). The test power, 1-β is also presented. p-value smaller than 0.001 is shown by 0^+.

Width (m)	1.3	2.0	3.0
Facing pedestrian versus e-scooter	p=0^+,<br>1-β=1.0, 1.0,<br>C.I.=[0.104 0.144].	p=0^+,<br>1-β=1.0, 1.0,<br>C.I.=[0.089 0.170].	p=0^+,<br>1-β=1.0, 1.0,<br>C.I.=[0.252 0.328].

Table 4: Welch's test (two-way t-test for unequaled variances and sample sizes) results when an e-scooter faces a pedestrian versus when it overtakes a pedestrian, boxplots in Fig. 2(c): p-value and Confidence Interval (C.I.). The test power, 1-β is also presented. p-value smaller than 0.001 is shown by 0^+.

Width (m)	3.0
Facing versus overtaking a pedestrian	p=0^+,<br>1-β=1.0, 1.0,<br>C.I.=[-0.802 -0.653].

Table 5: Welch's test (two-way t-test for unequaled variances and sample sizes) results when an e-scooter faces a single pedestrian versus when it faces a pedestrian in a crowd, boxplots in Fig. 2(d): p-value and Confidence Interval (C.I.). The test power, 1-β is also presented. p-value smaller than 0.001 is shown by 0^+.

Width (m)	2.0	3.0
Facing an isolated pedestrian Vs. a pedestrian in a crowd	p=0.018,<br>1-β=0.92, 0.52,<br>C.I.=[-0.155, -0.016].	p=0.070,<br>1-β=0.64, 0.42,<br>C.I.=[-0.006, 0.135].

Table 6: Welch's test (two-way t-test for unequaled variances and sample sizes) results when an e-scooter faces a single pedestrian versus when it faces a pedestrian in a crowd when the width changes, boxplots in Fig. 2(d): p-value and Confidence Interval (C.I.). The test power, 1-β is also presented. p-value smaller than 0.001 is shown by 0^+.

Width (m)	Facing a single pedestrian	Facing a pedestrian in a crowd
Widths 2.0 m and 3.0 m	p=0^+,<br>1-β=1.0, 0.99,<br>C.I.=[-0.160 -0.074].	p=0.45,<br>1-β=0.12, 0.11,<br>C.I.=[-0.05 0.122].

Table 7: Welch's test (two-way t-test for unequaled variances and sample sizes) results when an e-scooter overtakes a single pedestrian versus when it overtakes a pedestrian in a crowd, boxplots in Fig. 2(e): p-value and Confidence Interval (C.I.). The test power, 1-β is also presented. p-value smaller than 0.001 is shown by 0^+.

Width (m)	3.0
E-scooter overtaking an isolated pedestrian Vs. a pedestrian in a crowd	p=0.07,<br>1-β=0.60, 0.44,<br>C.I.=[-0.016, 0.382].
