# Supplementary material for: Pedestrians' safety using projected time-to-collision to electric scooters
Source: Nat Commun. 2024 Jul 7;15:5701. doi: 10.1038/s41467-024-50049-x (PMC11228023; doi:10.1038/s41467-024-50049-x)
Supplement: Supplementary file 3 — Description of Additional Supplementary Files [file 41467_2024_50049_MOESM3_ESM.pdf]

Description of Additional Supplementary Files

File Name: Supplementary Movie 1
Description: Pedestrians' safety using projected time-to-collision to electric scooters-Methods-Experiment Setup
